# Nano-Encapsulated Essential Oils as a Preservation Strategy for Meat and Meat Products Storage

**DOI:** 10.3390/molecules27238187

**Published:** 2022-11-24

**Authors:** Sergio A. Ojeda-Piedra, María L. Zambrano-Zaragoza, Ricardo M. González-Reza, Claudia I. García-Betanzos, Samantha A. Real-Sandoval, David Quintanar-Guerrero

**Affiliations:** 1 Laboratorio de Procesos de Transformación y Tecnologías Emergentes de Alimentos, Facultad de Estudios Superiores Cuautitlán, Universidad Nacional Autónoma de México, Cuautitlán Izcalli CP 54714, Mexico; 2 Laboratorio de Posgrado en Tecnología Farmacéutica Facultad de Estudios Superiores Cuautitlán, Universidad Nacional Autónoma de México, Cuautitlán Izcalli CP 54745, Mexico

**Keywords:** lipid oxidation, antimicrobial effect, natural preservatives, nanotechnology

## Abstract

Consumers today demand the use of natural additives and preservatives in all fresh and processed foods, including meat and meat products. Meat, however, is highly susceptible to oxidation and microbial growth that cause rapid spoilage. Essential oils are natural preservatives used in meat and meat products. While they provide antioxidant and antimicrobial properties, they also present certain disadvantages, as their intense flavor can affect the sensory properties of meat, they are subject to degradation under certain environmental conditions, and have low solubility in water. Different methods of incorporation have been tested to address these issues. Solutions suggested to date include nanotechnological processes in which essential oils are encapsulated into a lipid or biopolymer matrix that reduces the required dose and allows the formation of modified release systems. This review focuses on recent studies on applications of nano-encapsulated essential oils as sources of natural preservation systems that prevent meat spoilage. The studies are critically analyzed considering their effectiveness in the nanostructuring of essential oils and improvements in the quality of meat and meat products by focusing on the control of oxidation reactions and microbial growth to increase food safety and ensure innocuity.

## 1. Introduction

Meat and meat products obtained after the slaughter of animals are important elements of human diets. Fresh untreated meat is stored under freezing or refrigeration conditions and then vacuum-packed or commercialized in modified atmosphere packaging. Meat products, in contrast, are obtained through some form of processing that involves additives such as condiments, acetic acid, salt, and phosphates, among others [1,2]. Because meat is rich in proteins, fats, carbohydrates, vitamins, and pigments, it is an ideal medium for the development and propagation of pathogenic and non-pathogenic microorganisms. This means that meat and meat products require conservation methods that inhibit microbial decomposition and control deterioration reactions, associated mainly with the processes of lipid and protein oxidation that are considered the most important factors in preserving meat and meat products [3]. Meat products—including poultry—have a high fat content that makes them highly susceptible to oxidation reactions that can generate off-flavors, undesirable odors, and nutrient loss [4]. Lipid oxidation is affected by light, temperature, pH, and the saturation grade of fatty acids in meat. Moreover, meat proteins are susceptible to oxidation, producing changes in color and nutrient content [5]. Therefore, oxidation reactions and microbial growth are the principal mechanisms of spoilage and deterioration of food safety in meat and meat products, associated with the loss of flavor and color, perceptions of rancidity, and nutrient loss [6]. One way to avoid or prevent oxidative and microbial degradation consists in using chemical additives; however, these are considered unsafe and may be associated with the risk of developing cancer. For this reason, there is a growing interest in replacing those additives with natural products, such as plant extracts and concentrates, that can be applied directly to meat and meat products in their packaging. Especially important in this field, are natural additives with antioxidant capacity [7].

Essential oils (Eos) can be derived from numerous aromatic plants. Today, several Eos are being used in applications in food, cosmetic, and pharmaceutical industries, due to their antioxidant and antibacterial properties. In addition, EOs are generally recognized as safe (GRAS), so they can be used as additives in meat and meat products. The EOs most intensively studied are oregano, rosemary, thyme, mint, peppermint, clove, and eugenol. EOs are composed of alcohols, aldehydes, phenylpropanoids, ketones, terpenes, sesquiterpenes, flavonoids, and polyphenols [8], a fact that makes them subject to degradation under certain environmental and processing conditions. Nanoencapsulation is one method used to protect these compounds, increase their solubility, compatibility, vectorization, enhance the release of volatile and non-volatile components that confer antimicrobial and antioxidant properties, and permit controlled release. This process allows the compounds to act during processing or storage through direct application to meat and meat products or incorporation into packaging materials. The goals, of course, are to prolong shelf life and ensure food safety [9].

The application of nanotechnology in the food industry is defined as using submicron size materials (1–1000 nm) in various geometric forms (nanotubes, nanofibers, nanospheres, nano-cubes, among others). These nanostructures are used to entrap EOs in lipidic or polymeric materials to protect and stabilize them and control their release in the product [10]. Nano-encapsulated essential oils have been used in the food industry, especially for their usage in fruit and vegetable conservation. Some of these products could be used on meat and meat products for their antioxidant and antimicrobial capacity. A nanoemulsion with lemongrass essential oil developed by Oerlikon Balzers Coating AG is used for food packaging with gas barrier activity [11]. Another commercial nanoparticle systems with possible meat application are “Nano Tea”, used in the beverage industry by Shenzen Become Industry & Trading Co., and “Conservante Aquasol” by Aqua Novaa, used for the nanoencapsulation of lipophilic food additives [12]. This review considers recent advances in the development of nanosystems to protect EOs and obtain their controlled release to decrease microbial growth and oxidation reactions in meat and meat products. In Table 1 was presented a list with the abbreviations used in this research.

## 2. Mechanism of Deterioration in Meat

Meat is an important source of nutrients in human diets because of its high-quality proteins, essential amino acids, and B-group vitamins [13]. 

However, numerous physical and chemical factors make meat and meat products highly perishable. Some features to consider for proper processing and storage are high water activity (aw), pH, and nutrient content. Together, these elements create an excellent medium for the proliferation of microorganisms [14]. The most common problems with spoilage in meat and meat products result from the ease with which they are affected by high rates of lipid oxidation and microbial contamination if they are not correctly handled and/or preserved. These processes deteriorate the quality parameters of meat and can potentially impact public health. The main preservation methods for these products are refrigerated or frozen storage, but a great deal of research is being done into methods for increasing storage periods and prolonging shelf life without affecting quality parameters. These efforts include incorporating exogenous antioxidants, usually synthetic in nature [15]. 

### 2.1. Lipid Oxidation in Meat

Lipid oxidation is the second-most important decomposition process in meat and meat products after microbial growth. Several problems can occur along the cold meat chain, especially inadequate temperatures or temperature variations during transportation or storage that affect quality parameters and reduce shelf life due to spoilage. Meat and meat products require strict conditions of temperature control to prevent lipid composition because they are so sensitive to oxidation and peroxide production [16]. Lipids are key components of meat that are responsible for several desirable characteristics, including flavor and aroma profiles, tenderness, and juiciness [17]. 

Lipid oxidation reactions consist of a series of free radical reactions between fatty acids and reactive oxygen species (ROS) that produce a form of oxidative degradation called rancidity. These reactions foster the formation of intermediate free radicals and final non-desirable products (e.g., thermodynamic stables) that can interact with other constituents of food, such as proteins, sugars, pigments, and vitamins, to negatively alter the critical properties of meat: color, texture, and nutritional value. They also contribute to the development of rancid off-flavors and aromas [18,19]. Certain products of lipid oxidation (for example, malondialdehyde, acrolein, 4-hydroxy-trans-nonenal, 4-hydroxy-trans-hexanal, and crotonaldehyde-like compounds) can cause diseases, from inflammation to atherosclerosis. They can also interfere with signaling pathways, leading to significant biomolecular damage, cell aging, and cancer [19].

Endogenous and exogenous factors influence lipid oxidation in meat and meat products. Fatty acid profiles and the presence of ROS produced by cellular metabolism are the principal internal promoters of oxidation. Other endogenous components that catalyze ROS formation and increase oxidation ratios are high water activity and the presence of transitional metals such as free iron, enzymes, and heme-proteins (for example, hemoglobin and myoglobin [18,19]). The main external factors that participate in meat oxidation are, processing temperatures, moisture, light, and atmospheric oxygen [17]. 

The process of lipid oxidation can be divided into three types: autoxidation, photo-oxidation, and enzyme-catalyzed oxidation, depending on the precise reaction mechanisms involved. Autoxidation is the most common form. It occurs via free radical chain mechanism in an autocatalytic manner. These radical reactions take place in three phases: initiation, propagation, and termination [19]. 

**Figure 1:**
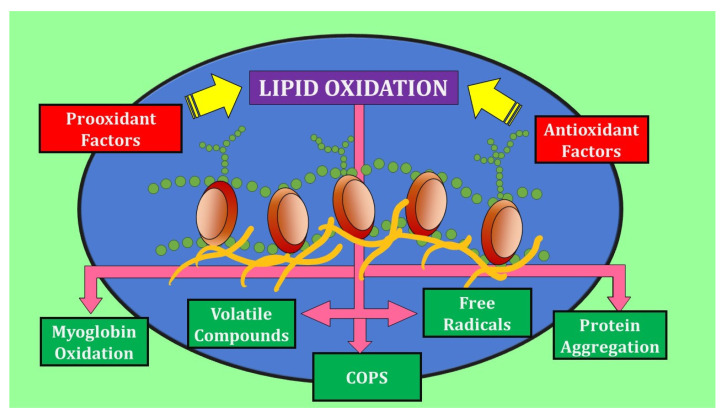
Schematization of lipid oxidation reactions in meat and the resulting changes.

Photo-oxidation is facilitated by radiant energy, mainly ultraviolet, in the presence of sensitizers such as meat myoglobin. It results in the production of hydroperoxide radicals (•OOH). In contrast, oxidation generated by the catalyzation of lipoxygenase enzymes leads to the formation of peroxides and hydroperoxides with conjugated double bonds [19]. Figure 1 presents a summary of lipid oxidation reactions. 

The first two phases of oxidation lead rapidly to the formation of lipid radicals and their subsequent transformation into non-radical compounds, such as conjugated dienes and hydroperoxides, which are considered primary products. These decompose into carbonyl compounds, such as ketones, alcohols, and aldehydes, known as secondary lipid oxidation products [15]. During initiation, ROS like the hydroxyl radical (•OH) or superoxide radical anion (O_2_^−^) extract a hydrogen atom from the methylene group of polyunsaturated fatty acids. Lipid radicals may form and then be rearranged through diene conjugation to stabilize the fatty acid radicals. Later, during propagation, these radicals react with atmospheric triplet oxygen (^3^O_2_) to generate peroxyl (lipid) radicals (LOO•) with high reactivity that extract a hydrogen atom from an adjacent polyunsaturated fatty acid to form a primary oxidation product called lipid hydroperoxide (LOOH). Propagation is followed by a series of secondary reactions that generate lipid degradation and the development of oxidative rancidity products. This continues into the termination phase, where the unstable peroxyl radicals become stable, non-radical products [15,17,18,19].

Because ROS have extremely short half-lives, measuring them directly is challenging, so several secondary products of lipid oxidation are measured instead. The thiobarbituric acid reactive substances (TBARS) assay is used to reflect the degree of lipid oxidation through a secondary oxidative product of malonaldehyde (MDA). MDA reacts with thiobarbituric acid to produce a red color that can be detected calorimetrically. It is one of the most widely studied secondary products of meat oxidation because of its carcinogenic and mutagenic activity caused by interaction with proteins and DNA. TBARS scores are expressed as milligrams of MDA per kilogram of the sample [15,20,21].

### 2.2. Protein Oxidation in Meat

**Figure 2:**
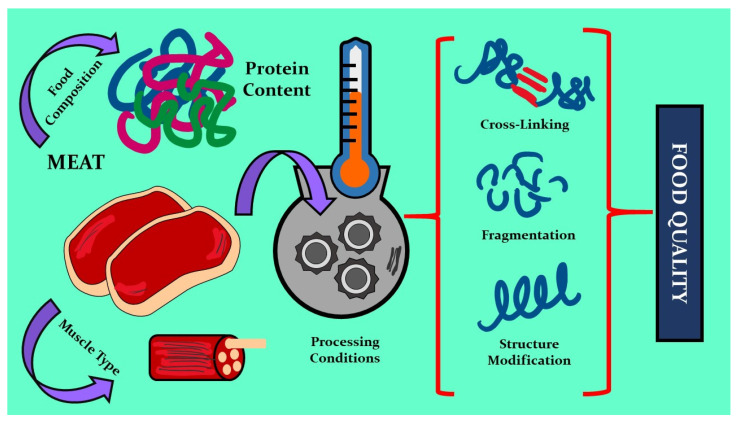
Changes in protein structure and food quality in meat.

Protein oxidation depends not only on lipid oxidation but also on intrinsic and extrinsic factors related to meat. Intrinsic factors include the animal species, the origin of the specific animal, muscle type, and natural components of meat such as unsaturated lipids, heme proteins, transition metals, and oxidative enzymes that can catalyze the formation of ROS. Extrinsic factors include processing and packaging conditions [22,23]. Figure 2 schematizes the protein modifications that occur during the processing and storage of meat and meat products.

ROS generate structural and conformation changes in proteins that result in protein crosslinking, modifications of amino acid side chains, protein fragmentation, and spatial protein rearrangement [15]. In addition, certain amino acids, primarily cysteine, tyrosine, phenylalanine, tryptophan, histidine, proline, arginine, lysine, and methionine, are easily converted into carbonyl derivatives that are especially susceptible to ROS [23,24].

The protein backbone and functional groups of amino acid side chains are common targets for radicals that react through hydrogen extraction to form carbon-centered free radicals (C•) that are subsequently converted into alkyl peroxy radicals (COO•) through the action of ROS. The radicals formed can react with another susceptible protein molecule to form alkyl peroxides (POOH). Further reactions with ROS, or reduced forms of transition metals such as Fe^2+^ or Cu^+^, lead to the formation of alkoxyl radicals (R-O•) and their hydroxyl derivative (POH), which trigger an oxidation chain reaction. Finally, under anaerobic conditions, the presence of (C•) leads to the emergence of carbon-carbon interactions (C•-C•) and cross-linked derivatives [15,23,25]. The modification of amino acid side chains reduces bioavailability and nutritional value, thus increasing the possibility of health damage due to the potential mutagenic, carcinogenic, and neurotoxic activity of protein oxidation and products, such as heterocycle proteins [22,24].

These structural modifications reduce susceptibility to digestive enzymes, while high oxidation rates result in cross-linking, massive aggregation, and the modification of protease-active sites. Protein oxidation also leads to the deterioration of physicochemical properties [22,24]. Two of the most important changes that occur are altered meat texture and increased muscle hardness due to protein cross-linking and the creation of disulfide bonds through the oxidation of cysteine thiol groups [25].

Myoglobin is a globular protein found in muscle. It contains a prosthetic heme group that gives both, myoglobin itself and its derivative compounds their distinctive red color. Myoglobin, however, is highly susceptible to oxidation that produces a brown-colored oxidative state that consumers associate with spoilage. The oxidation of ferrous-oxymyoglobin (Fe^2+^) to ferric-metmyoglobin (Fe^3+^) is responsible for discoloration in meat. The change in the visual appearance caused by this oxidative process in muscle involves a transition from a bright red to a dull brown color with decreased redness (a* parameter) and increased yellowness (b* parameter) on the L* a* b* color scale [25]. 

The use of exogenous antioxidants as synergic conservation mechanisms, complemented by refrigerated or frozen storage, is common in processes designed to develop highly-efficient antioxidant and anti-microorganism systems to prevent spoilage during the conservation of meat. These compounds can be added directly to products in the form of food additives, or to animal feed for bioaccumulation in fatty tissue. Antioxidants are categorized by the synthetic or natural source from which they are derived [17,19,26].

Oxidative processes also alter the ability of proteins to bind with water molecules through hydrogen bonding, electrostatic repulsion, or capillary action. Consequently, the effect of protein oxidation on the water-holding capacity of meat involves a balance of promoting (e.g., increased myofibril swelling, negative net charges) and inhibiting factors (e.g., cross-linking or proteolysis reduction). In this case, both types of factors are dependent on the degree of protein oxidation [15,25]. Other functional parameters, such as protein solubility, gelation, and emulsifying properties, may also be modified. For example, high levels of protein oxidation decrease protein solubility through denaturation and precipitation. In contrast, protein oxidation can improve gelation and emulsification properties. The main explanation for the improvement of gel-forming ability with moderately-oxidized proteins relates to the increased formation of cross-linked structures between proteins that can stabilize other non-covalent bonds, thus decreasing the mobility of the gel network [23].

### 2.3. Meat Spoilage Due to Microorganisms

Because it is a raw material, meat is susceptible to quality deterioration during processing, especially if mishandled. In addition, the high water-activity characteristic of meat (~0.99) makes it an excellent medium for microorganism proliferation [14,27]. Common pathogens in meat include *Campylobacter jejuni*, *Clostridium perfingens*, *Escherichia coli* O157:H7, *Listeria monocytogenes*, *Salmonella*, *Staphylococcus aureus*, and *Campylobacter* spp. Biological contamination is one of the principal causes of the many food-borne illnesses that occur worldwide [15,28] and the primary reason behind the ongoing search for alternative methods that improve or ensure the safety of meat and meat products for consumers. EOs constitute an excellent option because, depending on their volatile composition, they can reduce the risk of microorganism growth in fresh meat, while combatting the development of degenerative proteolytic microorganisms like Pseudomonas and Acinetobacter, that generate undesirable changes in the color, texture, flavor, and odor of meat and meat products [29].

Like oxidation reactions, microbial growth in meat also depends on intrinsic and external environmental factors. Storage temperature is considered critical in reducing microorganism growth rates by maintaining temperature conditions below the optimal range for bacterial proliferation, which is 20–40 °C for most bacteria. Studies have established that anti-spoilage conditions to impede or prevent bacterial growth require constant storage temperatures below 4 °C [27]. Most of the microorganisms present in meat cannot grow at such low temperatures, though some –*Listeria monocytogenes*, for example– are psychrophilic, so they are capable of surviving and replicating slowly even under refrigeration temperature (≤7 °C), though optimal growth occurs between 20–30 °C. This is a main concern associated with processed meat products. Freezing is recommended to decrease water activity in meat and modify the environment required for microorganism growth by transforming water into ice and concentrating solutes in the remaining free water at levels that inhibit microbial growth [30,31].

However, groups of pathogens like *Salmonella* and *Staphylococcus* can survive even under frozen storage conditions. An important consideration related to frozen meat is that it behaves like its unfrozen counterpart during thawing, including high microorganism growth rates due to dripping that favors the development of psychrophilic organisms [31]. Finally, the effects of certain microorganisms that can contaminate innocuous tissues during slaughter and mishandling may endure despite adequate processing and storage conditions. Hence, incorporating antimicrobial agents is a key to meat preservation [27].

## 3. Use of Preservative Compounds with Meat and Meat Products

Preservation agents with antioxidant or antimicrobial activity used with meat and meat products must: (i) be environmentally friendly; (ii) not alter the organoleptic properties of products; (iii) not leave residues; (iv) not become a concern for public health; and (v) have GRAS status [29,30]. The study of agents with potential applications in the handling, processing, and storage of meat and meat products is an important field of research. Several preservation agents are described briefly in the following sections.

### 3.1. Synthetic Preservatives

Although synthetic antioxidants may have high efficiency even at low concentrations, their repercussions on human health are currently considered a significant downside by consumers due to the potentially severe health risks of carcinogenicity and diseases of the gastrointestinal tract. As a result, there is a movement towards replacing synthetic antioxidants with compounds of natural origin. The most common meat antioxidants currently used are butylated hydroxyanisole (BHA), butylated hydroxytoluene (BHT), propyl gallate (PG), and tert-butyl hydroquinone (TBHQ). BHA, BHT, and TBHQ, are synthetic chain-breaking antioxidants with aromatic rings that can donate a radical (H•) to an oxidizing lipid to block the oxidation chain reaction. PG is an aromatic antioxidant with three -OH groups on the phenyl ring that can donate three radicals [17,19,32]. The U.S. Food and Drug Administration (FDA) has established limits on antioxidants added to foods. For BHA, BHT, TBHQ, and PG, the general limit is 0.02% of fat or oil (alone or combined) in foods, including EOs [33].

### 3.2. Preservatives of Natural Origin

Natural origin preservatives are an alternative to conventional antioxidants. Considered safer for human health, these components enjoy greater consumer acceptance. However, in some cases, they must be used at high concentrations to achieve the same effect as commercial additives, an aspect that may cause undesirable variations in products, but can also have positive effects on the sensory properties of meat and meat products. This means that their use must be contemplated in combinations with other technologies to achieve optimal activity at minimal concentrations [34]. Several natural preservative agents with high antioxidant content are sourced from plants, including certain fruits, teas, herbs, nuts, spices, vegetables, and algae. The main chemical agents of natural antioxidants are phenolic compounds (anthocyanins, flavanols, flavones), carotenoids, essential oils, tannins, lignin, phenolic acids, and vitamins. However, these antioxidants are subject to losing their activity under adverse conditions, such as high temperatures, extreme pH, and the presence of light [17,19,32].

### 3.3. Antioxidants

Antioxidants are biological or chemical compounds that delay or prevent oxidation reactions by acting as reducing agents, free radical scavengers, or radical species inactivators. They can be classified as primary and secondary antioxidants. The former act as free radical scavengers that donate an electron or hydrogen atom to peroxyl or alkoxy radicals to prevent them from reacting with a new fatty acid, thus blocking the branching reactions that occur during autoxidation. The latter, often called hydroperoxide decomposers, convert hydroperoxides into non-radical, non-reactive, thermally stable products [18,19].

Animal tissues contain numerous initiators and components of catalytic oxidation, but also an endogenous antioxidant enzyme system that includes superoxide dismutase (SOD) and glutathione peroxidase (GPx), substances that can control oxidation processes by decomposing and eliminating ROS and other reactive oxygen species radicals. For example, GPx can control the hydrogen peroxides and lipo-peroxides formed during lipid oxidation. These enzymes continue to be active even in post-mortem muscle tissue and play an important role during meat processing and storage by preventing the oxidation of lipids and proteins and diminishing rancidity [35]. However, these systems have not yet demonstrated a sufficient capacity to delay oxidation in meat and meat products. Therefore, many strategies conceived to reduce oxidation include adding exogenous antioxidants [26].

### 3.4. Antimicrobials

Today, synthetic antibiotics are the antimicrobial treatments most often used with meat to inhibit pathogen contamination. They have, however, raised environmental concerns and an emerging awareness of the dangers of microbial resistance and its adverse effects on human health. The development of natural-origin antimicrobial systems based, for example, on EOs, as an alternative preservation system for meat and meat products has demonstrated the potential to replace antibiotics [30,36,37,38]. Moreover, global trends in food preservation, especially for meat and meat products, are heading towards bio-preservation; that is, the use of compounds of natural origin, including EOs. This movement has led to intensive efforts to develop green, eco-friendly extraction methods, standardization, and stabilization with the goals of obtaining reproducible results and high antimicrobial capacity.

## 4. Essential Oils

EOs are secondary metabolites obtained from plants, composed of complex mixtures of volatile, lipophilic, and aromatic compounds with low molecular weight. EOs can be obtained from many plant materials: flowers, buds, roots, bark, leaves, seeds, peels, fruits, stems, and wood. Many, however, are characterized by a strong odor [39,40]. 

These economic oils contain mixtures of elements that allow their use as agents to improve the sensory aspects of foods and beverages. They have a varied content of bioactive compounds that can function as antimicrobial and antioxidant agents, so they have potential for use as natural food preservatives [40]. Several beneficial effects are attributed to the terpenes, alcohols, acetones, phenols, acids, aldehydes, and esters present in EOs. Benefits for human health include anti-carcinogenic, anti-diabetic, anti-inflammatory, and anti-mutagenic activity [39,41]. However, a significant challenge to the use of EOs is that their composition and functionality depend on multiple elements: the source plant; its origin and specific characteristics; growth conditions (climate, hydration, and soil nutrients, among others); extraction conditions; and the methods utilized. Their use, therefore, always requires processes to standardize their composition to ensure that they have reproducible properties from batch-to-batch. Despite these drawbacks, the high antioxidant, and antimicrobial capacities, of many EOs are important properties that we describe in the following sections.

### 4.1. Antioxidant Capacity

As natural antioxidants, EOs have several action mechanisms that function to slow oxidation reactions. Phenolic compounds are among the most predominant components of several EOs. In some cases, they constitute up to 85% of the total composition. Carvacrol, eugenol, and thymol, are some of the best-known phenolic compounds employed in meat preservation. They can be defined as primary or chain-breaking antioxidants. Their hydroxyl groups (-OH) act as hydrogen donors to inactivate ROS during the initiation stage and impede the propagation of lipid-free radicals. However, EOs also contain secondary antioxidants with metal chelation activity over transition metals and free radical scavengers. Examples are ethylenediaminetetraacetic acid (EDTA) and citric acid, which chelate iron and copper ions to prevent metal-catalyzed lipid oxidation. Carotenoids absorb energy from singlet oxygen and transfer it to triplet oxygen with no change in chemical structure. They also protect EOs from light-induced oxidation [18,34].

### 4.2. Antimicrobial Activity

EOs have antimicrobial effects on diverse microorganisms, including bacteria, fungi, and viruses. Terpenoids, phenylpropanoids and phenols are the predominant compounds that are part of EOs composition with antimicrobial activity by their hydrophobic nature, being carvacrol, thymol, α-pinene, p-cymene, 1,8-cineol, D-limonene, γ-terpinene and terpinen-4-ol some of the most common [41].

Due to differences in their cell wall composition, gram-positive bacteria are usually more sensitive than gram-negative types. The latter have a thinner but more complex cell wall that gives greater resistance to natural hydrophobic extracts. In addition, they have an outer membrane with a high percentage of lipopolysaccharides that make it almost impermeable to lipophilic compounds. As a result, these bacteria can protect cell integrity by resisting the infiltration of EOs active compounds. The hydrolases contained in the periplasmic space between these two membranes aids in degradation. The cellular structure of gram-positive bacteria, in contrast, allows hydrophobic molecules to penetrate the cytoplasm relatively quickly, allowing lipophilic molecules direct contact with the phospholipid bilayer of the cell membrane [29,42]. 

Therefore, the antimicrobial activity cannot be attributed to a single effect; the primary mechanism of EOs action against microorganisms is associated with the lipophilic behavior that permits interaction with the hydrophobic components of the cell membrane to foster the bioaccumulation of EOs in the membranes of microorganisms, where they interact with the peptidoglycan structure of the cell wall to inhibit synthesis, thus generating cell wall damage. Structural modification causes the loss of membrane integrity, alters permeability, and induces changes in the functioning of the electron transport chain, nutrient absorption, and the outflow of macromolecules [40,42,43,44]. When bioactive compounds enter the cytosol, they can interact with protein and nucleic acid synthesis, promote the coagulation of cellular content, inhibit enzymatic activity, and alter respiration and energy metabolism. These agents also have the capacity to inhibit the growth and reproduction of pathogenic bacteria by blocking nutrient absorption and transport [42,43]. Phenolic compounds are mainly responsible for their microbial activity linked to the number and position of their hydroxyl group; in general, the more phenolic compounds the more antibacterial properties against foodborne pathogens [41].

Fungi are generally more resistant to antimicrobial agents. EOs exert an antifungal effect by disrupting the cell wall, altering cell membranes, coagulating cytoplasm, and, finally, damaging cellular organelles to promote leakage of the cellular contents. It has been suggested that lysis of the fungal cell membrane is caused by interaction with EOs and their binding to ergosterol, which is the main sterol present in fungal cell membranes, responsible for controlling permeability and fluidity [36].

## 5. Nanosystems for Encapsulation of Essential Oils

As mentioned previously, EOs contain unstable functional compounds that depend on environmental conditions, such as high temperatures, pH variation, light, and the degree of hydration. Variations in these parameters can alter the antioxidant and antimicrobial efficacy of EOs. These oils may interact with food matrices by binding to carbohydrates, fats, or proteins, but the effectiveness of this action may require a dose increase to achieve the desired effect. The challenge here is that larger doses may negatively impact the organoleptic properties of food products due to the intense sensory characteristics of EOs (flavor, odor, color), causing consumer’ rejection [36,41].

Nanotechnology is an emerging field of research that presents an option for maintaining the efficiency of EOs incorporated into meat and meat products. This is important because the direct incorporation of EOs into products can accelerate oxidation, rapidly reduce effectiveness, and cause the loss of volatile components due to their compatibility with the lipids in meat. In addition, some EOs produce sensory changes when added to products through polymerization reactions that interact with certain components of meat to modify their effectiveness [45]. The advantageous properties of nanosystems include their large contact surface, the nature of their interaction with components of meat and meat products, the potential to increase compatibility with food matrices, and their capacity for controlled release that maximizes effectiveness as antimicrobials or antioxidants during storage.

The methodologies utilized in the elaboration of nanosystems are generally classified as “top-down” or “bottom-up”. Top-down techniques use mechanical energy to reduce the size of the source material from the macro range, while bottom-up methods consist in forming smaller, dispersed molecules or particles through chemical or physical means of self-assembly [46,47]. Today, numerous reviews and book chapters are available on the preparation methods of different nanosystems, including nanoemulsions, nanofibers, polymeric nanoparticles, and lipid nanocarriers, among others [48,49,50,51]. 

The European Commission defines nanomaterials as “natural, incidental, or manufactured materials containing particles, in an unbound state, as an aggregate or as an agglomerate, where at least 50% of the particles have one or more external dimensions in the size range” [52]. Nanomaterials are commonly categorized by their dimensional features as zero- (nanoparticles), one- (nanowires, nanotubes), two- (films, membranes), or three-dimensional structures (nanocomposites, dendrimers [53,54]). They can also be classified as inorganic (including metals, metal oxides, salts, clay) or organic (polysaccharides, proteins, lipids). The main nanosystems for which we have findings related to the preservation of meat and meat products are discussed in the following subsections. This review shows that a great deal of the information available focuses on the role of EOs in meat packaging. However, we also highlight advances in the incorporation of nanosystems as ingredients or preservatives in meat and meat products to reduce oxidation reactions and microbial growth and, thereby, prolong shelf life.

Metal nanostructured materials are the most commonly used in the food industry as antimicrobial-biocide agents and disinfectants for equipment, surfaces, and production rooms [55]. Their effectiveness depends on achieving a high surface-volume ratio and stability in processing conditions such as high temperatures and pressures. Their utilization in packaging materials allows the continuous release of active compounds during storage periods and, potentially, continuous microbial inhibition [36]. These materials are not recommended for applications that involve direct contact with foods, but they are used as reinforcing agents in the structure of food containers where they improve mechanical properties, modify permeability, and provide antimicrobial action. The metals most commonly used at nanoscale are silver (Ag), copper (CuO), and zinc (ZnO), incorporated in the form of metallic ions or metal oxides [36,55].

The action mechanism of inorganic nanomaterials proceeds through the release of ions that promote electrostatic interaction with microorganisms that damages the integrity of bacterial cells and impedes the formation of ROS. Certain metals, such as silver ions, form complexes with sulfur, phosphorus, nitrogen, and oxygen, in the functional structures of enzymes of microorganisms. This leads to the destabilization of the cell membrane and generates disturbances in microbial cellular metabolism [36]. However, inorganic nanoparticles also have drawbacks as they may cause physicochemical changes and raise the lipid oxidation index of food matrices by promoting the generation of ROS. A second concern is that metal nanoparticle applications that come into direct contact with foods may bioaccumulate with potentially toxic effects. Finally, when used in packaging materials, there is a danger that metal ions could migrate into food matrices [56]. 

A recent analysis of the interaction between inorganic nanoparticles and food matrices highlighted that this behavior depends on the precise composition of both materials. For example, polyphenols increase the toxicity of ZnO nanoparticles, but reduce enzyme activity. However, inorganic nanoparticles can also form a biocorona around proteins, carbohydrates, and enzymes, among other components of foods, which modifies the absorption and behavior of nanostructured additives, possibly reducing their activity and modifying their functionality [57]. In this regard, it is important to emphasize that inorganic nanoparticles are a good option for meat packaging materials. For example, a study using tilapia fish skin gelatin and ZnO nanoparticles with ginger essential oil showed good antibacterial activity against mesophiles and psychrophiles, as well as antioxidant action on lipid oxidation in the fish. In addition, modifications of the mechanical and thermal properties of the film applied were observed [58]. 

A study using ZrO_2_ nanoparticles and the EOs from *Zataria multiflora* in a biobased film that incorporated pectin/potato starch examined the shelf life of quail meat. The results showed that the film increased shelf life, while decreasing microbial growth [59]. The application of bimetallic nanoparticles (Ag-Cu) with cinnamon EO to chicken meat in the form of a polylactic film showed that those nanoparticles modified the transport properties and transparency of the film [60]. These findings suggest that combining EOs with inorganic nanoparticles represents a good packaging option for meat and meat products, one that can improve food safety and shelf life. 

In the rest of this review, we focus on systems that contain essential oils. Aspects such as the consequences and toxicology involved in the use of inorganic nanoparticles will not be addressed further.

### 5.1. Organic Nanosystems

**Figure 3:**
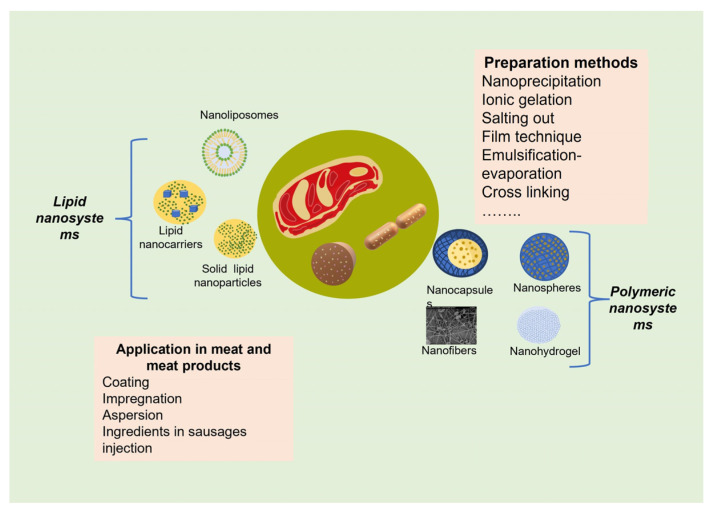
Nanosystems as preservatives in meat and meat products.

Organic nanomaterials used to preserve meat and meat products must be food-grade, preferably of natural origin, biocompatible, eco-friendly, and biodegradable [47]. The principal components of nanosystems are biodegradable polymers, biopolymers (proteins, polysaccharides), phospholipids, lipid that are solid at room temperature, and liquid lipids [53]. The nanostructures most often used as ingredients in foods, especially in the meat industry, are nanoemulsions, solid lipid nanoparticles (SLN), nanostructured lipid carriers (NLC), nanoliposomes, nanofibers, and nanohydrogels [61]. Figure 3 shows the main nanosystems used as components in meat and meat products. The organic nanoparticle systems have presented an outstanding progress for the effective delivery of bioactive compounds in specific sites and applications. Organic nanoparticulate systems are promising as active vectors due to their ability to release active substances, improve the stability of Eos, and can be biocompatible with tissues and cells when they are fabricated from biocompatible or biodegradable materials. The classification as controlled release systems are based on the mechanisms of release, having those controlled by diffusion, chemical impulses, osmotically controlled and swelling and/or controlled by dissolution [48].

#### 5.1.1. Nanoemulsions

Nanoemulsions are prepared with a lyophobic colloidal system composed of two immiscible liquids that, when dispersed, form globules measuring 20–500 nm [62]. Since EOs are insoluble in water and nanoemulsions are easy to prepare, they are an excellent alternative for incorporating natural preservatives into meat and meat products [45]. Likely due to the ease of incorporating nanoemulsions into food matrices, and their easy preparation and economic aspects, they are the nanosystems most often employed in meat processing, where they are used in coatings or as ingredients in the preparation of sausages, fresh meat, and marinated meat products. Their use has been reported mainly with poultry, beef, and pork. Studies have demonstrated that EOs improve the compatibility of nanoemulsions with meat and meat products and increase the shelf life of sausages and other products [63]. EOs have been incorporated into nanoemulsions to prepare edible coatings for ground beef using *Zataria multiflora* essential oil reinforced with cinnamaldehyde. Observations show that an edible coating containing this essential oil nanoemulsion had the best results in terms of decreasing lipid oxidation, while having only a minimal effect on sensory acceptability [64].

#### 5.1.2. Nanoliposomes

Structures formed with lipid walls commonly used with meats are called nanoliposomes. These are spherical vesicles formed by a bilayer of phospholipids around water. They are excellent carriers of hydrophilic and hydrophobic materials, have a large surface area, increase solubility, improve controlled release, permit targeting, and show good encapsulation efficiency [65]. The disadvantage of nanoliposomes is their instability. Nanoliposomes made with garlic EO have been used to increase the shelf life of beef hamburger patties. In that study, researchers evaluated garlic EO entrapped in nanoliposomes. The samples tested did not develop Salmonella and the normal amount of Staphylococcus aureus decreased significantly after 8 days of refrigerated storage. Moreover, the sensory qualities were similar to those of patties without garlic EO. Finally, the samples containing the nanoliposomes showed that nanoencapsulation helped to control the release of the essential oil during storage, thus prolonging shelf life [66]. 

#### 5.1.3. SLN and NLC

SLN are formed with a lipid that is solid at room temperature. The methods employed in their elaboration are high-energy emulsification, emulsification-evaporation, and microfluidization. One study of SLN loaded with cinnamon and ginger EOs prepared by solvent emulsification assisted by ultrasonication, and applied them as antioxidants in beef hamburger patties, showed that the antioxidant effect increased, accompanied by a reduction of TBARS value during frozen storage. These findings indicate lower lipid oxidation when EOs were released from the coating onto the surface of the patties [67]. 

NLC are another lipid nanosystem used to preserve meat products. A study that evaluated the antimicrobial effect of NLC incorporated with thymol (the main component of oregano, thyme, and rosemary EOs) on sausages inoculated with *S. aureus*, *E. coli*, and *C. perfringes* (10^5.5^ CFU/g) found that thymol alone and the thymol-NLC decreased the growth of microorganisms most effectively in sausages with added nitrite [68]. 

The use of lipid nanosystems is compatible with the lipids present in meat and meat products, so they have a good conservation effect when used as either an antioxidant or antimicrobial. Thus, it is possible to consider EOs as effective natural additives for meat and meat products. More examples of the addition of nanosystems to meats for their preservation are discussed below.

#### 5.1.4. Polymeric Nanoparticles

**Figure 4:**
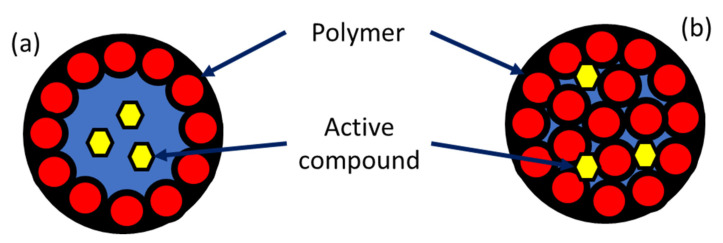
Nanoparticle structures: nanocapsule (**a**), nanosphere (**b**).

Biopolymeric nanoparticles are defined as submicron-sized solid colloidal particle systems that can include both nanosphere and nanocapsule structures. They have a polymeric matrix into which a compound can be loaded, encapsulated, or absorbed onto the surface [47,69]. Size distribution is the fundamental characteristic of these nanoparticles, as they have an upper limit of 1000 nm due to the weaker nature of their interaction compared to that of inorganic nanomaterials [53]. The main difference between the two possible structures lies in their architectural design. Nanocapsules are composed of a solid, hollow polymer shell (Figure 4a) surrounding an internal cavity or core where an active ingredient can be loaded. Alternatively, compounds can be placed in the liquid or solid phase of a dispersion. Nanospheres, in contrast, are composed of a dense matrix with no membranes or external layers (Figure 4b). In these structures, the active ingredient can be dissolved and permanently-, temporarily-, or covalently-linked to the polymeric matrix [70]. 

The encapsulation of EOs into polymeric nanostructures improves their physical and chemical stability, reduces highly volatile behaviors, and inhibits susceptibility to chemical degradation under environmental and/or process conditions [54]. The protection provided by coating agents blocks possible interaction with components of the food, but maintains bioactivity. Most of the polymers used to encapsulate EOs are hydrophilic because this allows a more homogenous dispersion of the oily lipophilic compounds in the mostly watery matrix of food products, thus increasing bioavailability and reducing the dose required to achieve the desired antioxidant and antimicrobial effects [53,54].

The nano-encapsulated active agent is released via the diffusion or erosion of the polymeric matrix. In both types of nanomaterials, release is influenced by solubility, diffusion rate, biodegradation of the matrix materials, and nanoparticle size [71]. The advantages of encapsulating EOs are that it can mask their characteristically intense odors and flavors, while increasing exposure to bioactive compounds and release times in the food matrix, all of which enhance efficacy. Adding polymeric layers permits the formation of modified and controlled release systems of the bioactive compound into the food matrix that can increase the shelf life of meat and meat products during storage [36,41,54,72,73]. Polymeric nanoparticles are the structures that have been studied most widely in relation to the encapsulation of EOs for applications in food preservation, including meat and meat products. For example, chitosan nanocapsules containing limonene, linalool, menthol, and thymol, were used with minced meat, where they reduced *E. coli* and peroxide production. Studies of this kind show that polymeric nanoparticles can be used to preserve meat [74].

#### 5.1.5. Nanofibers

Nanofibers are ultrathin, non-woven mats made from natural polymers that are utilized in packaging. They are usually used to entrap bioactive, nano-encapsulated compounds to enhance the antimicrobial and antioxidant activity of the resulting coating. They are fabricated from such biopolymers as polysaccharides to produce food packaging materials due to their large contact surface, porosity, degradability, and enhanced mechanical endurance [21,73]. In meat preservation, for example, glycyrrhiza-polysaccharide nanofibers loaded with tea tree EO have been used to preserve the microbiological quality of pork and poultry products with a reduction of 97.86% of Salmonella typhimurium growth. Moreover, the nanofibers helped reduce lipid oxidation—according to the quantification of TBARS—while maintaining texture and color [21].

#### 5.1.6. Nanocrystals

Nanocrystals for use in foods are prepared with starch and cellulose to obtain crystalline fractions. Acid hydrolysis is the method most often employed. Nanocrystals have excellent thermal resistance and stability. Their size is ≤100 nm. These nanosystems are the most innovative in the formulation and preservation of meat and meat products. They have been studied for their potential incorporation into Pickering meat emulsions to stabilize systems like sausages and dressings, and to mask the undesirable flavors of many EOs [75]. Regarding the application of nanocrystals in meat, edible coatings and packaging materials are focus areas for use with fresh meat to minimize the growth of acid-lactic bacteria and decrease lipid oxidation [76]. Starch nanocrystals and nanoparticles are increasingly being utilized in the development of new meat products because the starch is used as a binding agent for lipids and water in the preparation of low-fat products and other applications. Nanocrystals also exhibit excellent interaction and stability in the controlled release of EOs by limiting the volatility of components like monoterpenes, sesquiterpenes, aliphatic aldehydes, alcohols, and esters, and reducing the possibility that their aromas could mask odors caused by decomposition.

#### 5.1.7. Nanogels

Nanogels are carriers of bioactive compounds, including EOs. They are defined as nanometric particles that use water as a solvent during preparation. Nanogels prepared with proteins and polysaccharides can retain large volumes of water without losing their three-dimensional structure [77]. They are characterized by a mixture of the properties of hydrogels and nanomaterials that increases the bioavailability and biocompatibility of the EOs incorporated in them [78]. For example, chitosan nanogels cross-linked with benzoic acid have been used to encapsulate rosemary EO as an antibacterial agent in beef chops. Observations in that case showed that at a concentration of 2 mg/g of beef the rosemary EOs nanogel achieved better inhibition of Salmonella than the unencapsulated EOs, thus extending the shelf life of the refrigerated meat [79].

#### 5.1.8. Inclusion Complexes

Cyclodextrin has a hydrophobic center made up of a cyclic glucose oligosaccharide with bound α (1–4). Inclusion complexes were formed by α-, β-, and γ-cyclodextrin. The cavities produced had diameters of 0.57 nm, 0.78 nm, and 0.95 nm, respectively [80]. Due to their low toxicity and high compatibility, complexes of this type have been used to encapsulate Eos to increase the shelf life of meat [81]. However, inclusion complexes can have problems of instability and may be difficult to incorporate directly into meat products due to high water content that can destabilize cyclodextrin. Other reports show the use of cyclodextrins to encapsulate eugenol in internal pads for packaging lamb meat. Tests have been performed to evaluate cyclodextrins for their antioxidant and antimicrobial effects and potential use in meat preservation, but this substance is still subject to extensive research. An additional problem is that its cost may make it unfeasible as a natural ingredient added to meat and meat products [82].

## 6. Applications of Nanosystems in Meat Preservation

Nano-encapsulated Eos are often used in coating systems for meat preservation. In this case, the product is immersed in a medium containing the nano-encapsulated active compounds to create an edible biofilm or coating. Applications of nanofiber include fabricating material for wrapping meat as part of packing systems [83,84].

Thyme (*Thymus vulgaris*) essential oil is widely used to preserve meat due to its antimicrobial and antioxidant capacity derived from its content of phenols and terpenoid compounds. The main components of thyme EO are thymol (38.1%), 5-methyl-2-(1-methyl ethyl) phenol and carvacrol (29.1%), and 2-methyl-5-(1-methyl ethyl) phenol [85]. Reports suggest that thyme EO has antimicrobial activity on gram-positive bacteria and yeasts. Although the action mechanisms involved have not been fully elucidated, these compounds interact with the cell membrane to modify permeability and enzymatic activity, including energy production and the synthesis of essential metabolites [86].

Studies such as [87], for example, have developed stable thyme EO nanoemulsions entrapped in a chitosan matrix to generate a nanoparticle coating system (139.47 nm) to the preserve fresh pork refrigerated at 4 °C for 12 days. That nanoparticle system demonstrated better antimicrobial activity on gram-positive bacteria—e.g., *Staphylococcus aureus*—than on gram-negative bacteria, like *E. coli*, with inhibitory effects of 60.35 and 55.50%, respectively, on in vitro assays. In other results, after 12 days of storage, the total viable count (TVC) on pork samples was performed, resulting in a reduction of 12.79% in meat samples treated with a nanoparticle-based coating system. The coating also served to preserve the sensory parameters of the pork during storage. Measurements of the color parameters (a*, b*, L*), demonstrated low variation after 12 days of storage. 

Snoussi et al. (2022) [88] developed a polymeric matrix to enhance the encapsulation of thyme EO. Their nanoparticle system contained 40% chitosan, 40% gum Arabic, and 20% medium-chain triglycerides (110.19 nm). It proved effective as an antimicrobial and antioxidant coating system for beef, as after 20 days of storage at 4 °C, total coliforms, psychotropic bacteria, and yeast and molds decreased by 48.87, 20.21, and 12.83%, respectively, compared to uncoated samples. Significantly higher TBARS values were also observed (33%) in the control sample (*p* < 0.05). Table 2 summarizes other research on nano-encapsulated thyme EO as a coating system to preserve meat during refrigerated storage.

Other EOs studied for their potential use in meat preservation include tea tree EO (*Camellia sinensis*). In their study, Cai et al. (2021) [21] encapsulated tea tree EO in gliadin nanoparticles (375.62 nm) for incorporation into glycyrrhiza-polysaccharide nanofibers. The use of this treatment in a wrapping system for meat decreased Salmonella typhimurium (TVC) by 98.52% in pork samples and 97.86% in chicken samples after 5 days of refrigerated storage (4 °C), compared to untreated samples. The antimicrobial effect was attributed to the presence of terpinen-4-ol in this EO. In addition, an antioxidant effect was observed with this nanofiber application that reduced TBARS values by 70 and 60%, respectively, in the samples of chicken and pork, an effect possibly due to the presence of α-terpineol, terpinolene, and γ-terpineol. 

Another EO of great interest for applications with meat is derived from clove (*Eugenia caryophyllata*) because the sensory profile of this EO is highly compatible with meat flavor [92]. The characterization of the clove EO incorporated into a nanoemulsion prepared with corn and soybean oils to coat chicken fillets before refrigeration (4 °C) identified six compounds, predominantly eugenol (88.38%) and β-caryophyllene (6.01%). Compared to non-coated samples, the nanoemulsion-treated fillets presented a 17.95% reduction of TVC bacteria and 15.50% of psychrophilic bacteria, with a reduction in the TBARS value of 20%, and enhanced sensory properties. 

Nanoparticles can function in immersion dispersions to form edible coatings for meat preservation, though some researchers have also incorporated them into wrapping materials. Hemmatkhah et al. (2020) [93], for example, fabricated an active cellulosic paper (10 wt%) containing encapsulated cumin seed EO (*Cuminum cyminum*) to extend the shelf-life of ground beef. The bioactive compounds responsible for the functional properties of that paper were cumin aldehyde, p-cymene, D-limonene, γ-terpinene, and eugenol. The antimicrobial and antioxidant effects of the activated paper were evaluated after 7 and 60 days of storage at 4 °C (refrigerated) and −18 °C (frozen), respectively. They obtained reductions of the TBARS value of around 40 and over 10% during frozen and refrigerated storage, respectively. In addition, after refrigerated and frozen storage, reductions in microorganism development in total mesophilic counts reached 20 and 10%, respectively, and approximately 15 and 10%, respectively, on psychrophilic bacteria counts. Other studies with diverse EOs are summarized in Table 3. 

## 7. Conclusions

Since meat is a product with a high risk for oxidation reactions and the growth of pathogenic microorganisms that lead to product loss, there is a growing need for viable alternative preservation measures to minimize losses and prevent the development of food-borne illnesses. Currently, essential oils are an option for decreasing oxidation reactions and controlling microbial growth in meat; however, it is necessary to ensure that these oils do not alter the sensory qualities of products while exerting their antimicrobial and antioxidant effect to prolong product shelf life. Nanoencapsulation technology preserves the antimicrobial and antioxidant properties of essential oils, increases their physico-chemical stability, and modifies their release profiles. The antioxidant and antimicrobial capacities of nano-encapsulated essential oils have been tested and proven effective in meat preservation systems and in enhancing the quality parameters of meat and meat products during refrigerated and frozen storage. 

## 8. Future Perspectives

Coming years will see increased research on many essential oils to test their effectiveness in combatting several key problems of meat spoilage. Identifying, and then characterizing, the bioactive compounds of each EO are necessary steps in elucidating their antimicrobial and antioxidant mechanisms. Studies must determine the effect of individual secondary lipid and protein oxidation products to ensure the inhibition effect of EOs on compounds that could affect human health. Another important field of study will focus on developing new nanostructures composed of inorganic substances that reinforce the positive effects of EOs. Studies of the mechanisms and cellular interactions that endow products with antioxidant activity, especially on lipids in the cell membrane, and studies of the conservation of the fibrillar structures of meat, can be designed to improve inhibition of protein oxidation. It is also necessary to continue elucidating the action mechanisms that serve to maintain protein structures during storage using essential oils. The development of nanosystems is sure to continue, based on various EOs and using environmentally-friendly methods that are easily reproducible. These efforts will make it possible to obtain nanosystems that can be applied directly during meat processing and scaled up to the industrial level.

## Figures and Tables

**Table 1:** List of abbreviation used in the review.

Abbreviation	Meaning
BHA	Butylated hydroxyanisole
BHT	Butylated hydroxytoluene
EDTA	Ethylenediaminetetraacetic acid
Eos	Essential oils
FDA	U.S. Food and Drug Administration
GPx	Glutathione peroxidase
GRAS	Generally recognized as safe
MDA	Malonaldehyde
NLC	Nanostructured lipid carriers
PG	Propyl gallate
ROS	Reactive oxygen species
SLN	Solid lipid nanoparticles
SOD	Superoxide dismutase
TBARS	Thiobarbituric acid reactive substances
TBHQ	Tert-butyl hydroquinone

**Table 2:** Nanosystems used in meat preservation.

Nanostructure	Polymer Layer	Average Size Distribution (nm)	Product Sample	Results *	Reference
Nanoparticles	Chitosan	Not reported	Fresh pork	After 12 days of storage (4 °C) ≥20% TBARS ≥20% *Pseudomonas*	[89]
Nanofibers	β-cyclodextrin-ε-polylysine nanoparticles and gelatin nanofiber	206.2	Fresh chicken	After 6 days of storage (4 °C) 4% aerobic bacteria count ≥ 20% TBARS	[90]
Nanoparticles	Chitosan	121.03	Beef patties	After 8 days of storage (4 °C) ≥40% enterobacteriaceae ≥25% mesophilic ≥68% *Staphylococcus aureus* ≥67% yeast and mold ≥84% TBARS	[91]

* Percentage reduction when applying a nanoparticle coating system, compared to untreated samples before storage.

**Table 3:** Essential oil applications in meat preservation.

**Nanostructures**	**Polymer**	**Essential Oil**	**Active Compound**	**Particle Size (nm)**	**Product Sample**	Results *	**Ref.**
Nanoparticle	Chitosan	Mandarin (*Citrus reticulata*)	(−) limonene (78.89%) and γ-terpinene (14.56%)	161.9	Fresh pork fillets	After 6 days of storage (4 °C) ≥25% TVC bacteria	[94]
Nanoemulsion	Chitosan	(*Schizonepeta tenuifolia*)	Not reported	92.3	Fresh pork slices	After 16 days of storage (4 °C) 1.94 *Pseudomonas* spp. log cycles 1.87 *Enterobacteriaceae* log cycles, 50% TBARS	[95]
Nanoparticles Nanoemulsion	Pectin	Curcuma (*Curcuma longa)* & Ajowan (*Carum copticum*)	7-bis(4-hydroxy-3-methoxy-phenyl)-1,6-heptadiene-3,5-dione Thymol (59.8%) and γ-terpinene (18.9%)	10 nm curcuma nanoparticle (CNP) 90.23 nm	Lamb loins	25 days of storage (4 °C) Total mesophilic bacteria: ≥25% CNP, ANE ≥35% mixture (CNP & ANE) Total psychotropic bacteria: ≥30% CNP, ANE ≥40% mixture (CNP & ANE) *Enterobacteriaceae*: ≥35% CNP, ANE ≥50% mixture (CNP & ANE) TBARS: ≥37% CNP, ANE ≥60% mixture (CNP & ANE)	[96]
Nanoparticles	Whey protein	*Aloysia citrodora* (*geranial* 28.32%*, limonene* 12.59% and *arcurcumene* 8.63%)	Not reported		Chicken breast slices	After 12 days of storage (4 °C) ≥45% TBAR; ≥20% Mesophilic bacteria; ≥15% Psychotropic bacteria ≥13% *Pseudomonas ssp.*	[97]

* Percentage reduction when applying a nanoparticle coating system, compared to untreated samples before storage.

## Data Availability

Not applicable.
